# Supplementary material for: Method development and validation for rapid identification of epigallocatechin gallate using ultra-high performance liquid chromatography
Source: PLoS One. 2020 Jan 15;15(1):e0227569. doi: 10.1371/journal.pone.0227569 (PMC6961889; doi:10.1371/journal.pone.0227569)
Supplement: Attachment — Submitted filename: Revision PONE-D-19-27318.pdf [file pone.0227569.s001.pdf]

An Ultra-Performance Liquid Chromatography method for single-analyte quantification of Epigallocatechin gallate (ECGC) in green tea was developed. The idea of the work is clear and the method was properly validated, as it seems. However, there are several language inconsistencies and terminology mistakes in some points that must be clarified and corrected for a better understanding and evaluation of the scientific content. Hence, I would consider the paper for publication in PLOSONE provided that the authors conduct major revisions on the manuscript, as detailed below. Needless to say, that a major revision of the English and the analytical language is highly advised for acceptance of the final manuscript.

Major revisions:

1) Please revise the short title for “UPLC determination of ECGC” as quantitative analysis is presented in the manuscript.

2) Please revise all units according to the International System and make them consistent across the entire manuscript text and figures (especially figure 1). All units must be separated from values by a space, except for percentage in which the number must be close to the value. Beware that “1 gr” and “micron” are not the correct way of expressing units (Figure 1 and respective manuscript text). In this sense, make the formatting consistent and correct throughout the whole manuscript and in the figures. Please include values of *n* in brackets, as the number of determinations, whenever a relative standard value is given.

3) Authors seemed to have been rather careless about significant figures. All inaccuracies must be corrected. For instance, LOD and LOQ should bare a single or two significant figures at the most.

4) Please revise the term “therapeutic doses of ECGC” (first paragraph of the introduction section) and clarify the sentence that follows “EGCG’s pro-oxidation action is regarded as a mechanism for its protective functions including its antioxidant properties”. This presents to me as an inconsistency to me as dose distinguished pro and antioxidant actions.

4) A more solid explanation must be given concerning the purpose of developing of a single-analyte method for ECGC detection by chromatography. Accordingly, authors must revise the third paragraph of the introduction section and link it with the first sentence of the following, on page 3: “ Despite previous studies, there is a need to optimize a simple and rapid method to identify the ECGC compound using a simple chromatography technique using UPLC system”. Also, remove “compound” and “using UPLC system” from the sentence.

5) Still on page 3 (last paragraph), please write “to validate the method by establishing the characteristic analytical parameters” and “validation criteria were”. Also, the ICH guideline that has been applied must be referenced here.

6) In section 2.2, clarify what do authors mean by “pharmaceutical grade samples of ECGC”. Do they refer to calibration standards? In section 2.4, please clarify what the “diluent” was.

7) Before chromatographic analysis, a three-step sample preparation was employed. Please explain the need for this cumbersome procedure to extract ECGC. Also, I am afraid that some of the analyte may be lost in the filter immediately before analysis. Recovery percentages are quite high for such a multi-step procedure. Could the authors address this issue and clarify? Also, please add information on the syringe filter material.

8) In section 2.6.2, please re-phrase: "Linearity was evaluated by carrying out the chromatographic analysis of several ECGC standard solutions with increasing concentrations and by establishing the calibration plot of the response vs. concentration, which visually approximates a straight line." Also, "To determine linearity (…), ECGC stock solution was diluted in mobile phase, yielding standard solutions with concentrations of (…)".

9) In section 2.6.3, please restrict the description to the strategy that has been used in this work for accuracy assessment. It is rather confusing how this determination was performed. Please re-phrase starting by "In the present study, accuracy was determined by spiking (…)"

10) In section 2.6.5, please re-phrase as follows "the LOD is defined as the lowest concentration of analyte which can be reliably detected in the test sample."

11) Please replace the word "optimise" by "studied", "evaluated" or "investigated" whenever appropriate as no design of experiments was undertaken.

12) In section 3.1, replace "five such trials were conducted" by "set of chromatographic conditions were tested". The authors must elaborate further on the options taken to establish the five trials as a poor discussion is provided. For instance, many variables have been changed from trial 1 to trial 2, preventing the isolation of the motive for rejection. Was it the column length or the mobile phase composition? Also, in table 1, please write "Mobile phase composition" and "Column features" in the appropriate column. What do authors mean by "Baseline is not proper"? Was it unstable, too much noise?

13) Please eliminate section 3.2 and include the comments in section 3.1. Renumber the rest of the section, accordingly.

14) Concerning section 3.3.1, define "blank" and "placebo" appropriately. Please re-phrase the second sentence, as the idea is simply unclear.

15) Please explain the purpose of performing a mass spectrometry analysis (page 11). Also, I do not recall reading the specifics of the determination in the methods section, particularly the *m/z* values of the main transitions used for ECGC identification. Please add this information wherever appropriate.

16) The first three sentence of section 3.3.2 is just a repetition of the information of the respective experimental section. Please re-phrase them appropriately as matter of discussion. Please write $R^2 \geq 0.9991$ and $R^2 \geq 0.9998$. Also, in Figure 4a caption, re-phrase as "Calibration curve obtained between peak area and concentration obtained for

standard ECGC solutions upon linearity determination.” The same phrasing should be applied for figure 4b. Please add error bars to the figures and the uncertainty obtained the slope and the intercept values. Finally, authors should explain the criteria to select the test concentration in light of the biological contents of ECGC.

16) Regarding Robustness, I do not see how temperature effect was evaluated. Please clarify.

16) Re-write the first sentence 3.3.4. It is just cumbersome.

Minor revisions:

1) Please revise the whole text for typos and make sure the references are in the correct format and numbered throughout the text.

2) Please replace “same developed conditions” by “selected conditions” on page 10 (section 3.2) and whenever appropriate.

3) Abstract: Please correct “its auto-oxidation property may lead to toxicity”, write “linear correlation coefficient > 0.9996”, and remove average recovery.

4) Remove capital letters from buffer salts, reagents, laboratory material (except for brands or equipment models), and analytical parameters within sentences.

5) Figures and Tables: - In figure 1, remove all capital letters within the sentences and the respective caption. Add the meaning of AOAC to the respective caption and to the manuscript text. Correct “supernate” to “supernatant”, also in the main text.
- Erase the column for average recovery on Table 2. Please write “Recovery (%), recovery values, and RSD (%)” in all tables instead of % Recovery, percentage recovery, and % RSD, respectively.
- Please revise significant figures in Table 3 as those are not consistent and in agreement with method precision. Also, express RSD values with a single decimal figure in all cases.
