# Supplementary material for: Method development and validation for rapid identification of epigallocatechin gallate using ultra-high performance liquid chromatography
Source: PLoS One. 2020 Jan 15;15(1):e0227569. doi: 10.1371/journal.pone.0227569 (PMC6961889; doi:10.1371/journal.pone.0227569)
Supplement: Attachment — Submitted filename: PONE-D-19-27318 R2 Responses.docx [file pone.0227569.s002.docx]

**Review Comments to the Author**

**Reviewer #1:**

**Comment 1:** The third line of the abstract is worded poorly and needs to be rewritten. Many of the previous wording concerns have been addressed, but a thorough proofreading is needed in any revision.

**Response:** As has been suggested by the reviewer, the third line of the abstract has been rewritten (Page number 2) and in-depth proofreading of the whole manuscript was done in the revised manuscript.

**Comment 2:** From the previous review "Comment 9: Page 11, top of the page: The difference observed by mass spectrometry must be explained. A difference of 0.8 Da is significant. What is the cause of the difference ?"

Response: Generally, in mass spectra molecular ion is obtained as protonated and
deprotonated ions. And deprotonation may have been observed in the green tea. We
have used 4000 Qtrap which is not high-resolution MS.

This response is not adequate. A difference of 0.8 Da is easily observed on a QTrap 4000. (I agree a QTrap 4000 is not a high resolution instrument.) If the authors think there is a protanation/deprotanation reaction, it needs to be demonstrated and explained. Why is there a Dalton shift between standard solutions and real samples?

**Response:** The authors are thankful to the reviewer for the comment. We have repeated the MS analysis for green tea extraction sample for the identification of EGCG several times and the mass was found to be 459.0 which is similar to mass of the standard EGCG which was also found at 459.0. Hence we could confirm that the developed method is suitable for identification of EGCG (Page number 11).

**Comment 3:**: All the chromatograms should have the same time axis so that the comparison is easier to see by the reader. I understand the change in run times, but the data should all have the same time scale.

**Response:** As suggested by the reviewer, all chromatograms were given with the same time axis (Figure 2a, 2b and 2c).

**Comment 4:** Page 14, Section 3.2.6: The last part of this section is not clearly written. Further the calculated LOQ and LOD need to have units with the values.

**Response:** As suggested by the reviewer, the last part of the section 3.2.6 has been rewritten and also units were given along with the LOD and LOQ values (Page number 14).

**Comment 5:** There are still issues with significant figures in Tables 2 and 3. For example in Table 2, the concentration of a solution is listed as 50 ug/mL (1 significant figure), yet the recovered amount is listed as 50.06 ug/mL (4 significant figures). The recovered amount can not have more significant figures than the amount you started with, 1 significant figure. In Table 3, the number of theoretical plates is given to 0.01, this is beyond the precision of the measurement. Both tables need to be revised further.

**Response:** As per the suggestion of the reviewer, table 2 and table 3 were revised in the revised manuscript. With respect to table 3, all insignificant figures in the tables were corrected and the SD of all the factors were found within the significant ranges as suggested by the reviewer.
